# Effectiveness of intraprocedural dual-phase cone-beam computed tomography in detecting hepatocellular carcinoma and improving treatment outcomes following conventional transarterial chemoembolization

**DOI:** 10.1371/journal.pone.0245911

**Published:** 2021-01-29

**Authors:** Youngjong Cho, Sangjoon Lee, Sung-Joon Park

**Affiliations:** 1 Department of Radiology, Gangneung Asan Hospital, Gangneung-si, Gangwon-do, South Korea; 2 Department of Radiology, Kangwon National University Hospital, Chuncheon-si, Gangwon-do, South Korea; 3 Department of Radiology, Korea University Ansan Hospital, Ansan-si, Gyeonggi-do, South Korea; Texas A&M University, UNITED STATES

## Abstract

To investigate the effectiveness of intraprocedural dual-phase cone-beam computed tomography (CBCT) in detecting hepatocellular carcinoma (HCC) during conventional transcatheter arterial chemoembolization (TACE) and its effect on improving treatment outcomes. Between November 2018 and November 2019, data from 111 patients with unresectable HCCs (N = 263 lesions) were reviewed retrospectively. All patients had undergone baseline magnetic resonance imaging (MRI) scans within one month prior to the procedure. Both arterial-phase (AP) and delayed-phase CBCT images were acquired during all conventional TACEs. Each HCC detection rate when read by AP-CBCT and when read by dual-phase (DP) CBCT including both AP and delayed phase was compared with that of MRI, and the diagnosis of HCC was based on MRI. Additionally, the follow-up results concerning lipiodol uptake status and tumor response of the lesions detected only by AP-/DP-CBCT were analyzed and compared with MRI-only detected lesions. The overall sensitivity of DP-CBCT (94.7%) was significantly higher than that of AP-CBCT (89.0%) (*p* = 0.003). In particular, the rate of subcentimeter HCC detection by DP-CBCT was pronounced (91.5% vs. 80.3%) (*p* = 0.01). Lesions found only by DP-CBCT exhibited positive lipiodol uptake (n = 31/31; 100%) and showed complete or partial responses (n = 24/31; 77.4%) on follow-up CT imaging, while MRI-only detected lesions had less lipiodol uptake (n = 6/14, 42.9%) and complete or partial responses (n = 4/14; 28.6%) (*p ≤* 0.001). DP-CBCT imaging during TACE enabled better detection of HCCs than when using AP-CBCT alone, and AP- and DP-CBCT is superior to MRI in detecting chemoembolization-sensitive lesions. This resulted in increased detectability of HCCs and the achievement of better treatment outcomes.

## Introduction

Hepatocellular carcinoma (HCC) is the third leading cause of cancer-related death worldwide [1]. In patients with HCCs not eligible for curative surgery, transcatheter arterial chemoembolization (TACE) plays a major role, and has been proven to be an effective locoregional therapeutic option that delivers survival benefits in inoperable patients [2–4].

The most common cause of early recurrence after TACE has traditionally been the inability to identify small or occult tumors before treatment [5]. However, success in detecting these tumors is essential for achieving the best treatment outcomes. With recent advancements in imaging technology, operators are more able to detect small HCC lesions [6].

Two-dimensional digital subtraction angiography (DSA), the conventional imaging modality for TACE, often fails to depict HCCs, especially those of a small size [5, 7–9]. Cone-beam computed tomography (CBCT), a recent imaging technique for the acquisition of three-dimensional volumetric images using the flat-paneled detector of the angiography suite, has advantages over DSA in detecting tumors with better spatial resolution [10]. Furthermore, it can provide guidance for navigating tumor feeders [7, 9].

Some authors have suggested that single arterial-phase CBCT outperforms CT diagnostically [11]. Moreover, when additional delayed-phase CBCT was applied, its detection ability was similar to or better than that of MRI, the so-called “gold standard” for HCC diagnosis [5, 11–13]. However, the number of lesions analyzed was small and the significance of the lesions additionally detected by dual-phase CBCT was not well-explained in these prior studies.

In the present study, using a higher sample number, we evaluated the effectiveness of performing dual phase (DP) CBCT including additional delayed-phase CBCT during conventional TACE for detecting HCC lesions. Additionally, we also analyzed the treatment outcomes of lesions detected only by DP-CBCT and only by AP-CBCT, not by MRI.

## Materials and methods

### Patient selection

For this type of study, formal consent is not required; this study obtained Institutional Review Board approval from the institution, and the need for informed consent was waived (GNAH 2020-05-019). For this type of study, consent for publication of the findings is also not required.

This study was not supported by any funding, and the authors declare that they have no conflict of interest.

Between November 2018 and November 2019, data from a total of 111 consecutive patients with unresectable HCCs treated by conventional TACE were reviewed retrospectively. Eligible patients for inclusion included (1) those who sequentially undergone DP-CBCT imaging during conventional TACE procedures and (2) those who had undergone baseline MRI scans within one month prior to the procedure. A total of 263 HCCs were collected for the final analysis. All procedures were performed by one interventional radiologist (Y.C).

### Dynamic contrast-enhanced MRI protocols

For baseline study, a 3.0-Tesla MRI unit (Skyra2; Siemens Healthineers, Erlangen, Germany) was used. Breath-hold contrast-enhanced (Gd-EOB-DTPA, 1 mL/s; Primovist; Bayer Healthcare, Berlin, Germany) T1-weighted three-dimensional fat-saturated spoiled gradient-echo images (volume interpolated breath-hold examination sequence, TE/TR: 1.6/4.0; field of view: 370 × 370; matrix size: 312 × 416; receiver bandwidth: 360 Hz/Px; slice thickness: 3.0 mm; and flip angle: 12°) were acquired using a bolus-tracking technique (AP: 17 seconds; portal venous phase: 30 seconds; delayed-phase: 90 seconds; and hepatobiliary phase: 20-minute delay). Single-shot breath-hold gradient-echo diffusion-weighted echo-planar images were acquired with the following parameters: TE/TR: 55/6500 ms; flip angle: 90°; matrix size: 208 × 260; slice thickness: 5.0 mm; and b-value: 800 s/mm^2^.

### Conventional transarterial chemoembolization protocols

Conventional TACE with hepatic arteriography, DP-CBCT, and post-procedural CBCT imaging were performed on all patients. A 5-French selective angiographic catheter (RH catheter; Cook Medical, Bloomington, IN, USA) was used for the DSA. The tip of the catheter was placed at the common hepatic artery or proper hepatic artery and the iodinated contrast medium [Visipaque^™^ (iodixanol); GE Healthcare, Chicago, IL, USA] was injected at a rate of 3 mL/s for seven seconds while observing the anteroposterior projection. If the right or left hepatic artery originated separately, then selective angiograms were performed using the iodinated contrast medium at 2 mL/s for seven seconds.

After the DSA study, DP-CBCT images were obtained. AP-CBCT was used to track tumor-feeding arteries using the EmboGuide software (Philips Healthcare, Best, the Netherlands). Conventional TACE was performed as selectively as possible for all suspicious HCC lesions according to the findings of MRI or CBCT. If additional suspicious lesions that had not been reported on previous MRI scans were visualized on the DP-CBCT images, they were also regarded as treatment targets. Conversely, if the lesions diagnosed in the previous MRI scans were not visible in DP-CBCT, conventional TACE was performed at the segmental level.

Depending upon the size and number of tumors, we injected a mixture of 10 to 50 mg of doxorubicin hydrochloride (Ildong Pharmaceuticals, Seoul, South Korea) and 2 to 10 mL of lipiodol (Guerbet, Villepinte, France). Additional embolization with gelatin sponge particles (Caligel 150–350 μm; Hangzhou Alicon Pharmaceutical, Zhejiang, China) was performed for the complete embolization of tumor feeders. Finally, nonenhanced postprocedural lipiodol CBCT imaging was performed to confirm the status of lipiodol uptake.

### Intraprocedural DP-CBCT technique

All CBCT imaging was performed using the single angiography system (Allura Xper FD20; Philips Healthcare, Best, the Netherlands). After the DSA study, refer to the previous paper on the imaging technique [11], DP-CBCT images were acquired through two consecutive rotations with three- and 30-second delay times following the initiation of single-contrast injection. The patients held their breathing twice during the gantry rotation. The default values for each CBCT scan were as follows: rotation time, 12 seconds; rotation angle, 240° arc at 20°/s speed; and 318 projection images. The three-dimensional volumetric data had an isotropic resolution of 0.7 mm for a 250 × 250 × 193 mm field of view. The thickness of the reconstructed axial image was 1.8 mm.

Depending on the catheter tip position, 15 to 20 mL of undiluted contrast medium [Visipaque^™^ (iodixanol) 270 mgI/mL; GE Healthcare, Chicago, IL, USA] was injected. When the catheter tip was successfully placed at the proper hepatic or common hepatic artery, a total of 20 mL contrast medium was injected at a flow rate of 2 mL/s. In the case of hepatic artery variation, both hepatic arteries were selected separately with injections of 1.5 mL of contrast medium for 10 seconds.

### Image analysis

For diagnosing HCCs, MRI was considered to be the gold-standard modality for this study. The diagnosis was made according to the American Association for the Study of Liver Diseases 2018 practice guidelines. According to Liver Imaging Reporting and Data System (LI-RADS), definite (LR-5) and probable (LR-4) lesions were included and were analyzed by two experienced abdominal radiologists.

For DP-CBCT imaging performed during the procedure, the operator (Y.C) judged the feasibility of such and proceeded with the procedure. Postprocedural lipiodol CBCT images were evaluated by two interventional radiologists (Y.C and S-J.P), one of whom (S-J.P) did not participate in the TACE procedures. In the event of any discrepancy, a consensus reading was accomplished by mutual agreement. Findings suggestive of HCCs on DP-CBCT images in this study were defined as follows: (1) distinct nodular-enhancing lesions on the AP-CBCT images or (2) lesions with delayed rim enhancement or corona enhancement on the delayed-phase CBCT images. Nodular lipiodol stagnation with high attenuation on nonenhanced postprocedural CBCT images was defined as an example of positive lipiodol uptake.

To evaluate the importance of the presumed HCC lesions additionally identified by DP-CBCT over preprocedural baseline MRI, presumed HCC lesions only on DP-CBCT were compared with those lesions found only by preprocedural MRI, considering their size, vascularity, degree of lipiodol uptake, and three-month responses. Three-month follow-up CT images were analyzed by two experienced abdominal radiologists.

### Statistical analysis

Statistical analysis was performed as a comparison between AP-CBCT and DP-CBCT performed in 111 patients with 263 tumors. Because this study sought to evaluate the effectiveness of additional dual-phase CBCT imaging conducted during conventional TACE in detecting HCC lesions, we evaluated the sensitivity on AP- and DP-CBCT with MRI as the gold standard according to the lesion size (i.e., subcentimeter vs. 1 cm or more). A pairwise comparison was conducted between AP-CBCT and DP-CBCT. The overall detection sensitivities and respective detection sensitivities (subcentimeter and ≥ 1 cm) were then compared using the McNemar test.

We analyzed the characteristics of tumors that were positive in AP- or DP-CBCT but negative in MRI (false positive in view of MRI) and that were negative in AP- or DP-CBCT but positive in MRI (false negative in view of MRI). The tumor size, vascularity, lipiodol accumulation status, and short-term (three-month) tumor responses were compared using Fisher’s exact test between these lesions only found by AP-/DP-CBCT and the lesions found only by MRI. The Statistical Package for the Social Sciences (version 25.0; IBM Corporation, Armonk, NY, US) was used for all statistical analyses and *p* < 0.05 was considered to be statistically significant.

## Results

The data of 111 patients with a mean age of 66 ± 10.4 years (range: 46–90 years; 84 men and 27 women) were evaluated retrospectively. The pretreatment patient demographics and baseline characteristics are listed in Table 1, and the respective sensitivity values of AP-CBCT and DP-CBCT for detecting HCCs are shown in Table 2.

**Table 1:** Baseline characteristics of patients with HCC before treatment.

Baseline characteristics	Values
**Patients number (N)**	111
**Age, years (mean ± SD)**	66 ± 10.4
**Sex, n (%)**	
Male	84 (75.7%)
Female	27 (24.3%)
**Etiology, n (%)**	
HBV	70 (63.1%)
HCV	8 (7.2%)
Alcoholic	24 (21.6%)
Cryptogenic	9 (8.1%)
**Child–Pugh class, n (%)**	
A	95 (85.6%)
B	16 (14.4%)
**ECOG performance, n (%)**	
0	89 (80.2%)
1	20 (18.0%)
2	2 (1.8%)
**BCLC stage, n (%)**	
0	21 (18.9%)
A	43 (38.7%)
B	45 (40.5%)
C	2 (1.8%)
**Laboratory tests (mean ± SD)**	
AFP, ng/mL	158.3 ± 647.9
Albumin, g/dL	3.7 ± 0.5
Total bilirubin, mg/dL	0.97 ± 0.56
AST, U/L	40.4 ± 28.5
ALT, U/L	27.0 ± 30.4
PT, %	86.1 ± 13.6
PT INR	1.12 ± 0.14

SD, standard deviation; n, number; HBV, hepatitis B virus; HCV, hepatitis C virus; ECOG, Eastern Cooperative Oncology Group; AFP, alpha-feto-protein; AST, aspartate aminotransferase; ALT, alanine aminotransferase; PT, prothrombin time; INR, international normalized ratio.

**Table 2:** Sensitivity of AP-CBCT and DP-CBCT in detecting HCCs diagnosed using MRI.

	Total (N = 263)	Lesions < 1.0 cm (n = 71)	Lesions ≥ 1.0 cm (n = 192)
**Sensitivity**			
AP-CBCT (%)	**234/263 (89.0%)**	57/71 (80.3%)	177/192 (92.2%)
DP-CBCT (%)	**249/263 (94.7%)**^a^^**1**^	65/71 (91.5%)^a^^2^	184/192 (95.8%)^b^

HCC, hepatocellular carcinoma; AP, arterial-phase; CBCT, cone-beam computed tomography; DP, dual-phase.

^a^Values between AP-CBCT and DP-CBCT were significantly different (1, *p* = 0.003; 2, *p* = 0.01).

^b^Values between AP-CBCT and DP-CBCT were not significantly different (*p* = 0.07).

The overall sensitivity of DP-CBCT (94.7%) was significantly higher than that of AP-CBCT (89.0%) (*p* = 0.003). In addition, the sensitivity of DP-CBCT (91.5%) was higher than that of AP-CBCT (80.3%) when identifying subcentimeter HCCs (*p* = 0.01).

**Fig 1:**
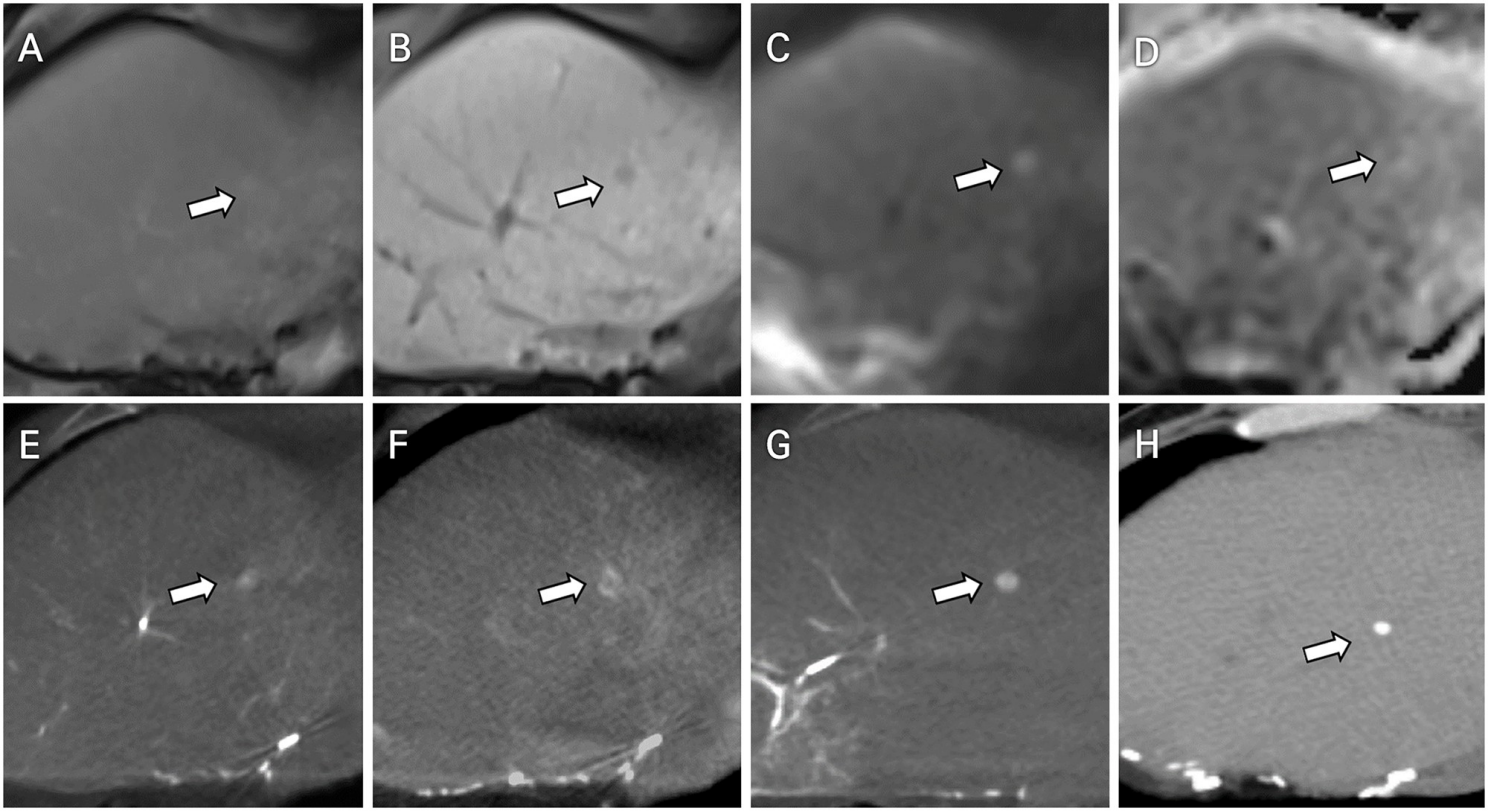
A 53-year-old male patient with right hepatectomy and recurred multifocal HCCs (Child–Pugh class A, BCLC stage B). (A) During contrast-enhanced MRI, a subcentimeter lesion (white arrow) in segment 4 remained nearly unidentified in the AP. (B) This lesion was seen as faint hypointensity on the hepatobiliary phase. (C and D) The tiny correlated lesion (white arrow) showed a high signal on DWI (b = 800) and low value on the ADC map, representing diffusion restriction. (E) AP-CBCT detected a faint hypervascular lesion in segment 4 (white arrow). However, upon interpretation, this lesion was recognized as an arterioportal shunt because of the indistinct margin. (F) This lesion was considered as a treatment target because delayed rim enhancement was noticed on the delayed-phase CBCT image (white arrow). (G) Positive lipiodol uptake was observed on the lipiodol CBCT image when chemoembolization was performed at the subsegmental level (white arrow). (H) Compact lipiodol uptake without residual arterial enhancement was visualized on the dynamic enhanced CT image scanned at the three-month follow-up (white arrow).

Among the 263 HCCs diagnosed using MRI, 11 lesions initially interpreted as arterioportal shunts on AP-CBCT images were later confirmed to be HCCs on delayed-phase CBCT images (Fig 1). Nine lesions unconfirmed using AP-CBCT due to motion artifacts were identified on delayed-phase CBCT images.

For HCCs measuring larger than 1 cm, DP-CBCT showed higher sensitivity over AP-CBCT (95.8% vs. 92.2%), but it was not statistical significant (*p* = 0.07).

**Fig 2:**
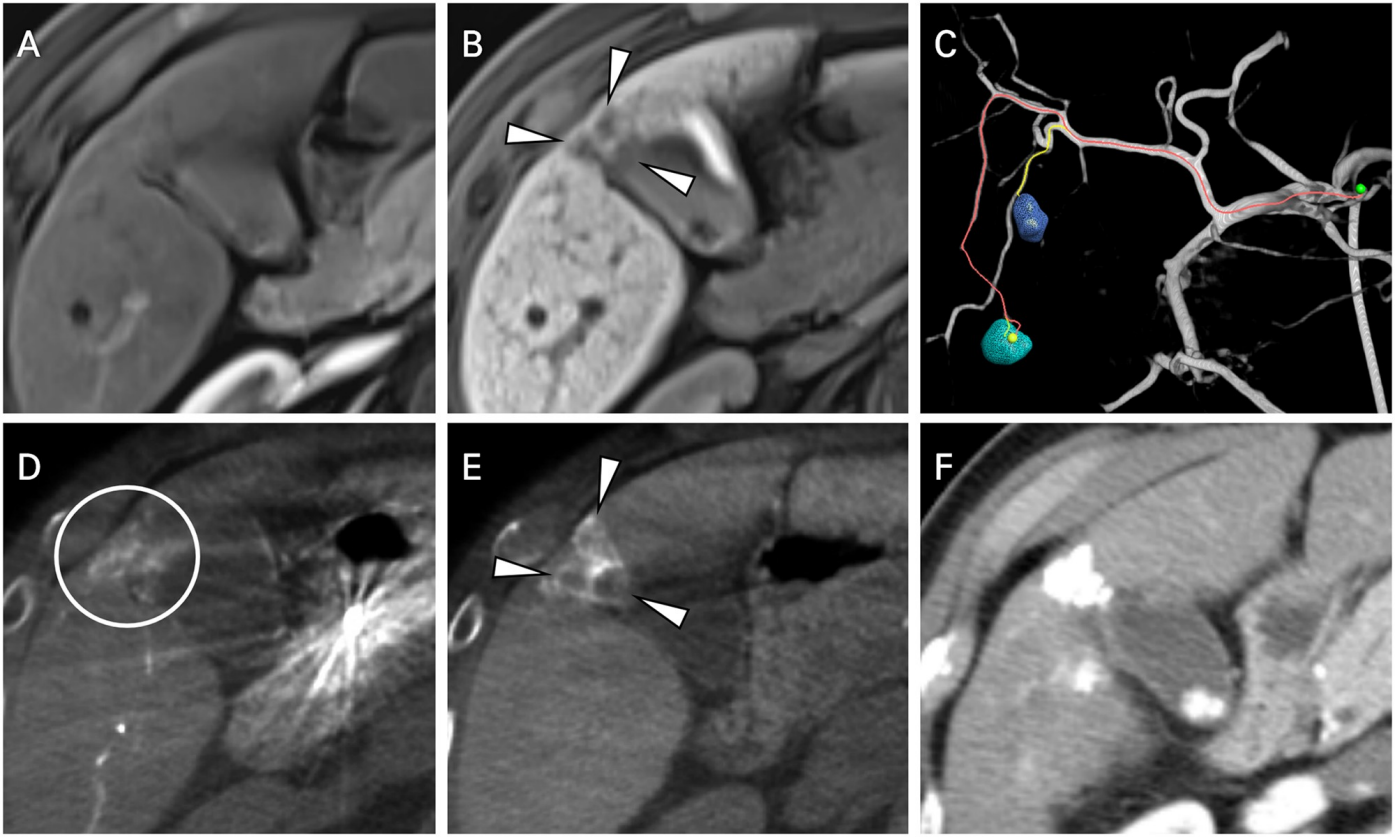
A 64-year-old male patient with recurred HCCs in segments 5 and 6 (Child–Pugh class A, BCLC stage B). (A) In the AP of MRI, there was no definite enhancing lesions at the gallbladder bed of the liver. (B) Three nodular hypodense lesions were identified in the hepatobiliary phase. These lesions were concluded to be indeterminate nodules upon reading (white arrowheads). (C) A preexisting tumor in segment 6 and its feeder are visualized with a yellow line by the EmboGuide^®^ program. An additional tumor (found by DP-CBCT) in segment 5 and its feeder are visualized with a red line. The treatment plan was changed during the procedure, with additional chemoembolization performed on segment 5. (D) Faint enhancement was visualized on AP-CBCT with an unknown degree of clinical significance (white circle). (E) Three rim-enhancing lesions were detected on delayed-phase CBCT (white arrowheads). (F) On three-month follow-up CT imaging, compact lipiodol uptake without residual arterial enhancement was visualized after TACE.

**Fig 3:**
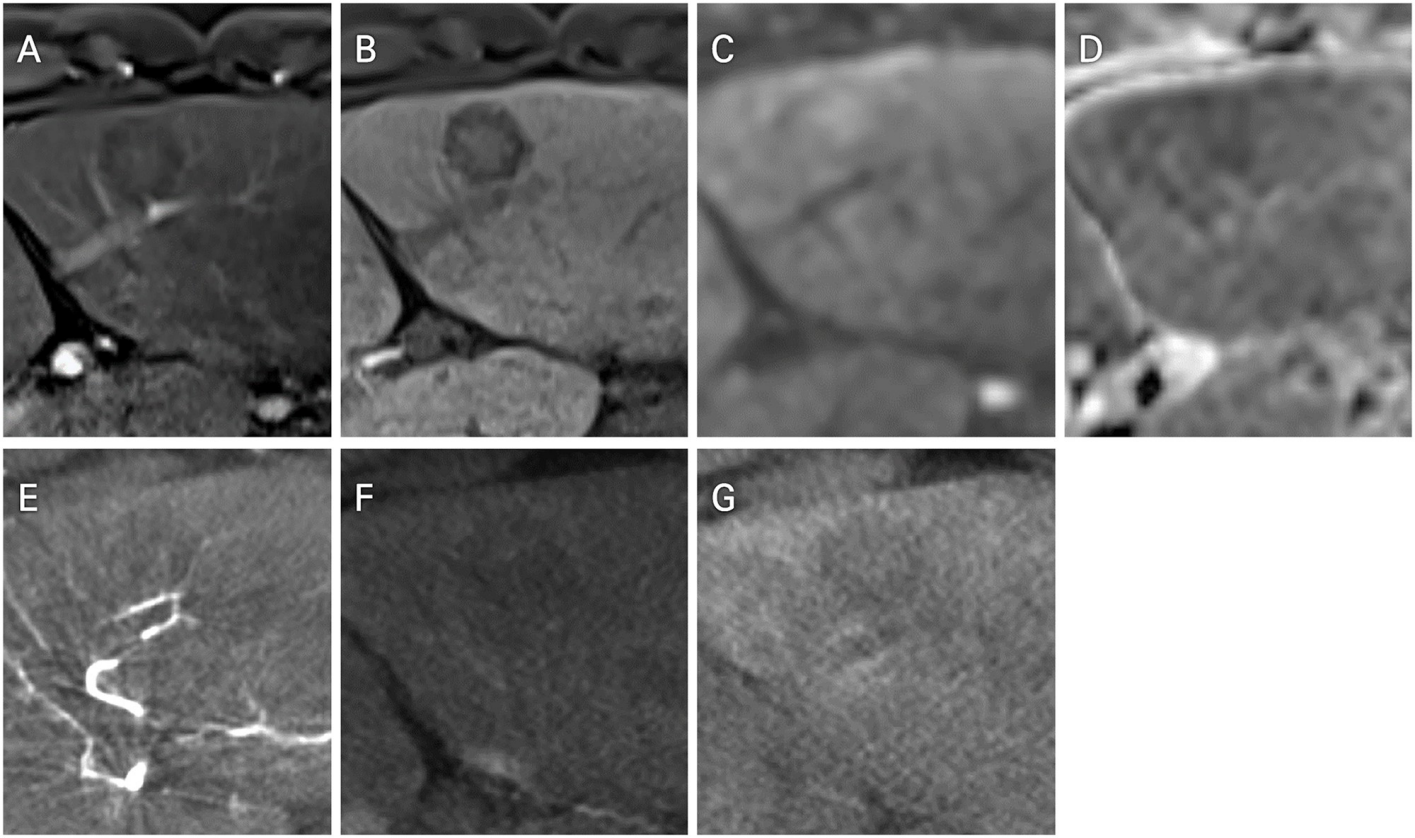
A 46-year-old male patient with two hepatocellular carcinomas (Child–Pugh class A, BCLC stage A). (A) A nonenhancing lesion of a low signal intensity (segment 3) was noted by AP-MRI. (B) Low signal intensity on the hepatobiliary phase and (C and D) diffusion restriction was also noted on DWI (b = 800) and ADC map. (E and F) No visible enhancement was noted by AP- or delayed-phase CBCT. (G) Negative lipiodol uptake was noticed after chemoembolization at the segmental level. This lesion was treated with radiofrequency ablation (not shown).

Furthermore, DP-CBCT suspected 31 more lesions to be HCCs than MRI did (n = 19 patients). Some of these lesions detected only using delayed-phase CBCT were also found in several sequences (e.g., hepatobiliary-phase or diffusion-weighted imaging) during MRI but did not meet the LR-5 or LR-4 criteria (Figs 1 and 2). This led to changes in the treatment strategy that we had planned only with preprocedural MRI (Fig 2). These patients also received additional subsegmental chemoembolization according to the positive findings of DP-CBCT. All patients receiving additional chemoembolization did not experience significant deterioration in their liver function. On the other hand, 14 lesions detected only using MRI were either not seen using DP-CBCT (Fig 3).

**Fig 4:**
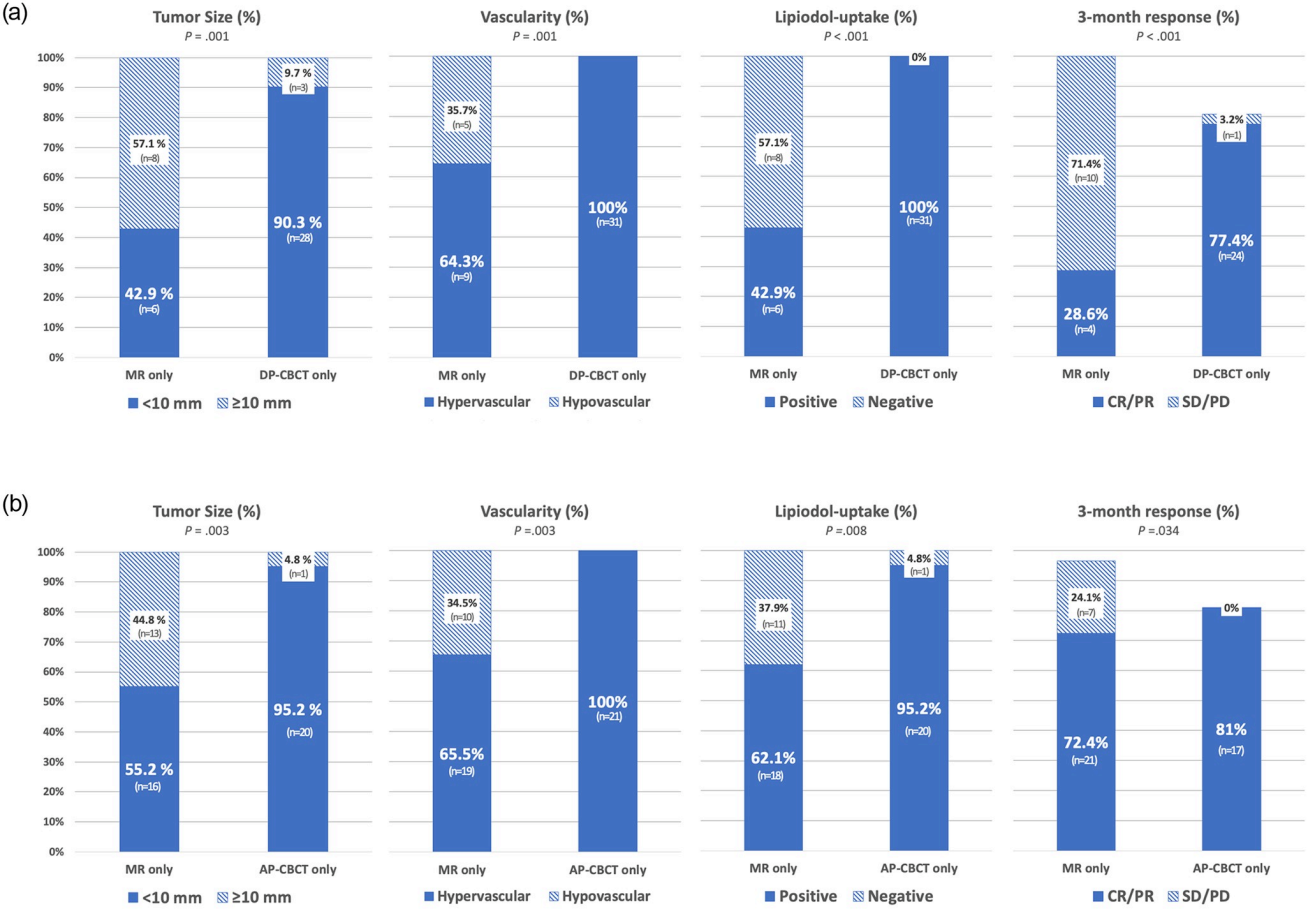
Comparison between lesions found only in CBCT and lesions found only in MRI. (A) Comparison between DP-CBCT (n = 31 in 19 patients) and MRI (n = 14 in 11 patients). (B) Comparison between AP-CBCT (n = 21 in 16 patients) and MRI (n = 29 in 25 patients).

Comparing the 31 lesions detected only using DP-CBCT and the 14 lesions detected only using MRI, respectively, DP-CBCT identified lesions that tended to be smaller and more hypervascular with more lipiodol uptake and with a better response following three-month CT imaging with statistical significance (Fig 4A). Importantly, all lesions found only on DP-CBCT images showed corona enhancement, reflecting hypervascularity and lipiodol uptake, during follow-up CT imaging. On the other hand, the response to conventional TACE among the lesions identified by MRI only was poor and treatment often involved another therapeutic option such as radiofrequency ablation. These findings were also seen on comparison between lesions found only by AP-CBCT and lesions detected only by MRI, but the difference in the proportion of positive lipiodol uptake was greater in the comparison between DP-CBCT and MR.

## Discussion

Two-dimensional DSA often fails to reveal small HCCs owing to the lack of vascularity in such lesions and the presence of patient motion artifacts [5, 7–9]. The identification of the tumor feeders of small HCCs is also difficult when they are overlapped by other vascular structures. CBCT, which provides three-dimensional volumetric images through a flat panel detector, has become a key technique for detecting tumors and providing guidance during TACE procedures [10]. As compared with multidetector CT, CBCT has a lower contrast-to-noise ratio (CNR) because of its lack of anti-scattering and easier susceptibility to motion, beam hardening, and ring artifacts. However, it achieves better spatial resolution owing to its smaller isotropic voxel size and the intra-arterial injection of the iodinated contrast agent compensates for the lower CNR to result in greater diagnostic performance. Research has shown that CBCT outperforms multidetector CT in the diagnosis of hypervascular HCCs and the identification of tumor-feeding branches [11]. Additionally, DP-CBCT has multiple roles in treating HCCs; notably with the addition of delayed-phase CBCT, hypervascular HCCs with corona enhancement can be detected [14] at a level comparable to that of MRI, which is regarded as the gold-standard imaging modality for HCC diagnosis [12]. In particular, this applies to small HCCs measuring less than 3 cm in diameter [5, 7, 9]. Recently, Lucatelli’s group reported that DP-CBCT can predict 1-month treatment outcome of the degradable starch microsphere-TACE intra-procedurally, and DP-CBCT shows better diagnostic performance than pre-procedural CT/MRI in TACE with liver cirrhosis [13, 15].

In this study, to evaluate the additional role of DP-CBCT, a pairwise comparison approach was used to compare the level of HCC detection between AP-CBCT and DP-CBCT among a greater number of cases than were included in previous reports [5, 12]. Additionally, the detection sensitivities were investigated according to differences in size (subcentimeter vs. 1 cm or more). The overall sensitivity of DP-CBCT outperformed that of AP-CBCT. This was likely due to variations in their capabilities for detecting smaller lesions (< 10 mm). Tiny enhancing lesions can be misdiagnosed as arterioportal shunts or overlooked on AP-CBCT. However, by adding delayed-phase CBCT, subcentimeter lesions with corona or delayed rim enhancements can be detected more reliably. As a result of hepatic vein occlusion during multistep hepatocarcinogenesis, the direction of tumor drainage is shifted to the adjacent hepatic sinusoids [16]. Contrast agents spreading to the nearby hepatic parenchyma can be visualized more easily based on the better CNR of delayed-phase CBCT, which aids in the location of these HCCs.

Because a finding of corona or delayed rim enhancement can be considered as suspected HCC, lesions with these findings on DP-CBCT were treated with conventional chemoembolization even though they did not meet the LI-RADS criteria on MRI. Interestingly, these lesions all showed compact positive lipiodol uptake results after TACE during three-month follow-up CT. A positive lipiodol uptake outcome can also be considered as a finding of malignant hepatocellular nodules [17]. An absence of the reticuloendothelial system and lymphatic system in the tumor as well as increased arterial flow and reduced portal flow lead to prolonged deposition of iodinated oil in the tumor [18]. Based on the hypothesis, clinically, lesions with lipiodol uptake newly seen on CT images taken several weeks after TACE can be judged as exhibiting tumor uptake. In previous papers, HCCs were defined based on lipiodol uptake alone or according to lipiodol uptake and suggestive image findings [17, 19, 20]. In our study, all lesions detected only by DP-CBCT exhibited positive lipiodol uptake (n = 31) when the lipiodol was injected at the subsegmental level, and 95.2% of all lesions detected only by AP-CBCT exhibited positive lipiodol uptake. Thus, a preprocedural MRI-based treatment plan could be changed during the procedure. For this reason, there was a phenomenon that the positive predictive value of DP-CBCT (88.9%) seemed to be inferior to that of AP-CBCT (91.8%) because there were more presumed HCC lesions with positive lipiodol uptake that were not detected by MRI in DP-CBCT (n = 31) than AP-CBCT (n = 21).

According to the modified Response Evaluation Criteria in Solid Tumor (mRECIST) criteria for the evaluation of tumor response after TACE, small lesions less than 10 mm in size are unmeasurable [21]. Therefore, the response evaluation in the subgroup analysis was limited because there were many subcentimeter lesions present. We categorized patients into a tumor-response group and a nonresponse group and found that the lesions detected by DP-CBCT showed a greater response to TACE than those found by MRI only; however, the impact on survival gain in light of this result requires further study.

Disadvantages to the addition of delayed-phase CBCT include radiation issues and lengthened procedure time. In the procedure of this study, efforts were made to minimize the number of DSA acquisitions and fluoroscopy time to reduce radiation exposure. Another disadvantage is that patients have to hold their breath twice during CBCT, which can cause discomfort and poor coordination. When artifacts were present, the image quality of CBCT fell significantly.

This study itself also had some limitations. First, histological confirmation was not performed for all the lesions. Instead, the diagnosis was based on imaging findings and lesions with rim enhancement only on delayed-phase CBCT were also defined as presumed HCCs in the subgroup analysis. This is somewhat different from the existing HCC imaging diagnostic criteria. However, among the results of subgroup analysis in this paper, lipiodol uptake was observed in all lesions with only rim enhancement, which paradoxically reveals limitations in the diagnosis of subcentimeter HCC according to existing imaging criteria. With further investigation, DP-CBCT could play an important role in the diagnosis of subcentimeter HCC. Second, there is currently no standard for DP-CBCT image acquisition. Third, this was a retrospective study.

## Conclusion

In conclusion, dual-phase CBCT during conventional TACE is more effective for identifying subcentimeter HCCs than AP-CBCT, and AP- and DP-CBCT is superior to MRI in detecting chemoembolization-sensitive lesions. The addition of this imaging modality is expected to result in improvements in the diagnosis and treatment of subcentimeter HCCs and better patient outcomes.

## Supporting information

S1 Data (XLSX)
