# Correction: Effectiveness of intraprocedural dual-phase cone-beam computed tomography in detecting hepatocellular carcinoma and improving treatment outcomes following conventional transarterial chemoembolization

**DOI:** 10.1371/journal.pone.0247648

**Published:** 2021-02-19

**Authors:** The PLOS ONE Staff

## Notice of republication

An incorrect version of Supporting Information file S1 Data was published. The publisher apologizes for this error. This article was republished on February 9, 2021 to correct for this error. Please download this article again to view the correct version.
